# Long Noncoding RNAs and Circular RNAs in the Metabolic Reprogramming of Lung Cancer: Functions, Mechanisms, and Clinical Potential

**DOI:** 10.1155/2022/4802338

**Published:** 2022-06-15

**Authors:** Yuhao Zhou, Yuan Zhan, Weiling Jiang, Huiguo Liu, Shuang Wei

**Affiliations:** Department of Respiratory and Critical Care Medicine, Key Laboratory of Pulmonary Diseases of Health Ministry, Tongji Hospital, Tongji Medical College, Huazhong University of Science and Technology, Wuhan 430030, China

## Abstract

As key regulators of gene function, long noncoding RNAs (lncRNAs) and circular RNAs (circRNAs) are generally accepted to be involved in lung cancer pathogenesis and progression. Recent research has clarified the phenomenon of metabolic reprogramming in lung cancer because of its significant role in tumor proliferation, migration, invasion, metastasis, and other malignant biological behaviors. Emerging evidence has also shown a relationship between the aberrant expression of lncRNAs and circRNAs and metabolic reprogramming in lung cancer tumorigenesis. This review provides insight regarding the roles of different lncRNAs and circRNAs in lung cancer metabolic reprogramming, by how they target transporter proteins and key enzymes in glucose, lipid, and glutamine metabolic signaling pathways. The clinical potential of lncRNAs and circRNAs as early diagnostic biomarkers and components of therapeutic strategies in lung cancer is further discussed, including current challenges in their utilization from the bench to the bedside and how to adopt a proper delivery system for their therapeutic use.

## 1. Introduction

Lung cancer (LC) is one of the most prevalent cancers and the leading cause of cancer-related deaths worldwide. Approximately 85% of LC cases are non-small-cell lung cancers (NSCLCs) [1]. Smoking (62.4%), ambient particulate matter (15.3%), and high fasting plasma glucose levels (9.9%) were the main reasons for disability-adjusted life-years with LC in 2019 for male and female patients [2]. During the past 20 years, significant progress has been made in LC pathogenesis. Notably, metabolic reprogramming, which is considered one of the hallmarks of cancer, has been confirmed to be heterogeneous and diverse. It was also confirmed to play a significant role in LC initiation and progression [3–5]. Moreover, it is worth noting that metabolic reprogramming is closely related to tumor proliferation, migration, invasion, metastasis, immune escape, and chemotherapy drug resistance [6–10]. Recently, progress has been made in the field of cancer metabolism. However, the exact molecular mechanisms involved are yet to be fully determined.

Long noncoding RNAs (lncRNAs) are a class of transcripts with lengths of more than 200 nucleotides that have essential roles in various biological processes of cancer cells, including metastasis, angiogenesis, and formation of the tumor microenvironment [11–13]. Circular RNAs (circRNAs) are formed by backsplicing events that involve a downstream 3′ splice donor site joining an upstream 5′ splice acceptor site in the primary transcript. Unlike lncRNAs, circRNAs have higher tolerance to exonucleases because of their covalently closed-loop structures with neither 5′ or 3′ polarity nor a polyadenylated tail [14]. The lncRNAs and circRNAs, which together have been found to have many binding sites for microRNAs (miRNAs), can participate in the regulation of key downstream target genes by acting as competitive endogenous RNAs (ceRNAs) for miRNAs. In this way, they trigger cancer pathogenesis and progression [15, 16].

**Figure 1:**
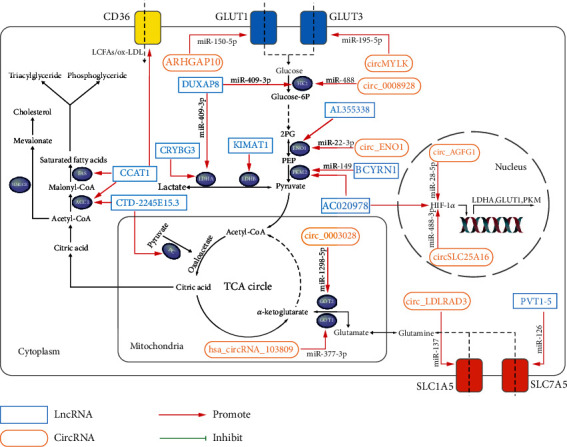
Important long noncoding RNAs (lncRNAs) and circular RNAs (circRNAs) regulate lung cancer metabolism by targeting key enzymes and protein mRNAs. *α*-KG: *α*-ketoglutarate; 2PG: 2-phosphoglycerate; ACC1: acetyl-CoA carboxylase 1; ENO1: *α*-enolase; FAS: fatty acid synthase; GLUT 1: glucose transporter member 1; GLUT 3: glucose transporter member 3; GOT1: glutamate oxaloacetate transaminase 1; GOT2: glutamate oxaloacetate transaminase 2; HIF-1*α*: hypoxia-inducible factor 1-alpha; HK2: hexokinase 2; HK: hexokinase; HMGCR: 3-hydroxy-3-methylglutaryl-CoA; LCFAs: long chain fatty acids; LDHA: lactate dehydrogenase A; LDHB: lactate dehydrogenase B; ox-LDL: oxidative low-density lipoprotein; PC: pyruvate carboxylase; PEP: phosphoenolpyruvate; PKM2: pyruvate kinase 2; SALL4: Sal-like protein 4; SLC1A5: glutamine transporter solute carrier family A1 member 5; SLC7A5: glutamine transporter solute carrier family A7 member 5.

**Figure 2:**
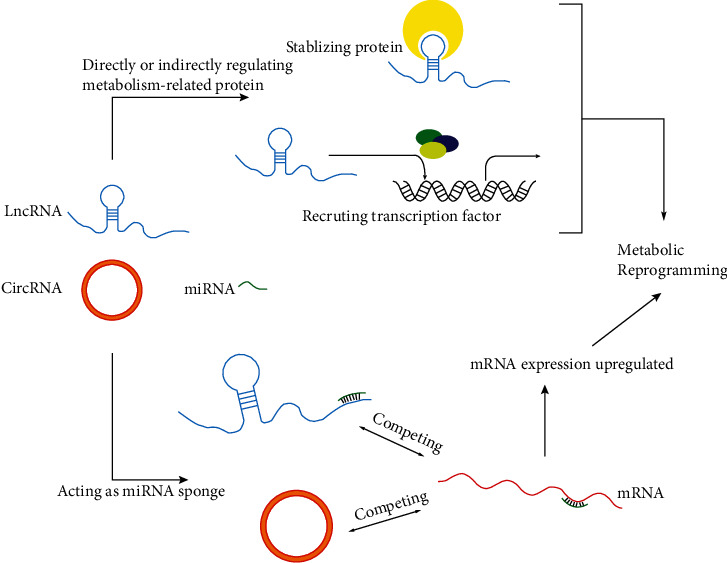
Molecular mechanisms of long noncoding RNAs (lncRNAs) and circular RNAs (circRNAs) in the metabolic reprogramming of LC.

Several recent studies have revealed the regulatory mechanisms of lncRNAs and circRNAs, mainly functioning as ceRNAs, in the pathogenesis of LC [17, 18]. We discovered that these mechanisms strikingly participate in the metabolic reprogramming of LC, thus significantly inducing the malignant phenotype of LC. This work discusses the regulatory mechanisms of lncRNAs and circRNAs in glucose, lipid, and glutamine metabolism in LC to provide deeper insight regarding LC pathogenesis and show potential therapeutic targets. The lncRNAs and circRNAs and their key target metabolism-associated proteins involved in LC metabolism are summarized in Table 1 and Figure 1. The specific molecular mechanisms of lncRNAs and circRNAs that have been discovered to regulate LC metabolism are summarized in Figure 2.

## 2. Glucose Metabolism

In contrast to normal cells, tumor cells heavily rely on aerobic glycolysis to produce energy and provide important metabolites to support their booming life activity despite sufficient oxygen supply. This distinctive characteristic has been generalized as the well-known “Warburg effect” by Otto Warburg [56]. The Warburg effect in tumor cells accelerates glucose uptake, causing nutrient deprivation in the surrounding normal tissue. It also provides materials for its lipid, nucleic acid, and amino acid metabolism, thus promoting its malignant behavior [57]; glucose transporter (GLUT) and several glycolytic enzymes, such as hexokinase (HK), pyruvate kinase (PKM), enolase (ENO), and lactic dehydrogenase (LDH), are aberrantly overexpressed in cancers, which contributes to metabolic reprogramming. They have been increasingly found to be novel therapeutic targets in cancers [58–62].

### 2.1. lncRNAs

#### 2.1.1. DUXAP8 and IGFBP4-1

The lncRNA DUXAP8 is overexpressed in LC tissues and cell lines and has been shown to induce proliferation, metastasis, and epithelial mesenchymal transition in NSCLC [63, 64]. Hexokinase 2 (HK2), which converts glucose to glucose-6-phosphate, is a key enzyme that participates in the initial and rate-limiting step of glycolysis. Lactate dehydrogenase A (LDHA) has an indispensable role of converting pyruvate to lactate in the final step of glycolysis. DUXAP8 sponges miR-409-3p to promote HK2 and LDHA expression, thereby promoting cell viability, migration, and glycolysis [22]. IGFBP4-1, another oncogenic lncRNA in LC, has been found to influence energy metabolism and promote expression of glycolysis-related enzymes such as HK2, LDHA, and pyruvate dehydrogenase kinase 1 (PDK1) [23]. However, the mechanism remains to be further studied.

#### 2.1.2. BCYRN1

As a key rate-limiting enzyme involved in tumor glycolysis, pyruvate kinase (PK) catalyzes the conversion of phosphoenolpyruvic acid and ADP to pyruvate acid and ATP. PKM2, one of the four isoforms of PK found in humans, is upregulated in tumor cells and cancer stem cells; it plays a crucial role in glycolysis and tumor malignancy [65]. The lncRNA BCYRN1 acts as an oncogene in NSCLC. Lang et al. observed that BCYRN1 increased expression levels of PKM2 and further induced glycolysis in NSCLC cells via the miR-149/PKM2 axis [25]. However, evidence of these significant findings is insufficient because of the lack of in vivo xenograft assays.

#### 2.1.3. CRYBG3

CRYBG3 was recently found to be a potent tumor-promoting lncRNA that is involved in inducing aneuploidy and promoting tumor metastasis in NSCLC [66, 67]. LDHA, a major subunit of LDH, catalyzes the last step of aerobic glycolysis, and its expression and activity reflect the intensity of tumor glycolytic activity. It is also involved in early carcinogenesis and tumor progression. Chen et al. reported that CRYBG3 could directly interact with LDHA to enhance the activity of the latter, thereby promoting aerobic glycolysis and proliferation in NSCLC cell lines [28]. However, the specific mechanism of their interaction warrants further investigation.

#### 2.1.4. KIMAT1

KRAS is an important oncogene in various cancers; mutant KRAS induces an increase in the expression of GLUT1, glycolysis, and mitochondrial respiration [68]. As a cellular energy sensor and modulator, AMP-activated protein kinase (AMPK) behavior in tumorigenesis remains largely unsolved. One study reported that an AMPK*α*-modulated lncRNA, KIMAT1 (ENSG00000228709), which is activated through KRAS mutation, regulates AMPK*α* activation by stabilizing lactate dehydrogenase B (LDHB) in NSCLC [27]. In contrast to LDHA, LDHB catalyzes the oxidation of lactate to pyruvate, which forms fuel energy for cells in the Krebs cycle, thus contributing to metabolic reprogramming and malignant biological behaviors of cancer cells [69].

#### 2.1.5. LINC00243

Serving as a gatekeeper of the tricarboxylic acid (TCA) cycle by inactivating pyruvate dehydrogenase and pyruvate dehydrogenase kinase 4 (PDK4), LINC00243 has a significant role in tumor metabolism. However, the expression of PDK4 varies greatly in different tumors, and its tumor-promoting and tumor-inhibiting effects are still unclear. LINC00243, a tumor promoter, is aberrantly increased in human NSCLC and has been associated with poor prognosis. LINC00243 promotes glycolysis and proliferation in NSCLC cells at the molecular level by positively regulating PDK4 through miR-507 sponging [34].

#### 2.1.6. AC020978

Unlike pyruvate kinase isozyme M1 (PKM1), which is generally expressed in highly differentiated cells, pyruvate kinase isozyme M2 (PKM2) is aberrantly expressed in metabolically active and poorly differentiated cells. PKM2 plays a crucial role in aerobic glycolysis in cancers by transforming phosphoenolpyruvate into pyruvate acid. The lncRNA AC020978 has been found to be upregulated in NSCLC, and its expression is increased with advanced TNM stages and associated with a significantly poor prognosis for patients. AC020978 was demonstrated to specifically bind with PKM2 protein, which enhanced its stability and induced its nuclear translocation. PKM2 that enters the nucleus forms a complex with HIF-1*α*, which promotes the latter's transactivation activity, leading to upregulation of the expression of AC020978 and glycolysis-related protein. Altogether, the AC020978/PKM2/HIF-1*α* signaling pathway formed a positive feedback loop, which greatly accelerates glycolysis and promotes NSCLC carcinogenesis [26]. Thus, AC020978 may be a promising biomarker for determining LC severity; the AC020978/PKM2/HIF-1*α* axis could be an underlying therapeutic target of LC in the future.

#### 2.1.7. LINC01123

As a well-known human oncogene that contributes to multiple hallmarks of cancer, c-Myc is a crucial transcriptional factor. Early studies indicated that transcriptional targets of c-Myc participate in many cell life activities in tumors, including metabolism, cell cycle, and apoptosis [70]. One study found that lncRNA LINC01123 expression was strongly raised in NSCLC tissues, which predicted an unfavorable prognosis. Importantly, researchers have demonstrated that LINC01123 enhances the metabolic reprograming of NSCLC cell lines in vitro. Moreover, the transcription of LINC01123 was confirmed to be directly initiated via c-Myc's transcriptional activity. Interestingly, LINC01123 could in turn upregulate the expression of c-Myc via ceRNA activity by sponging miR-199a-5p. Additionally, LINC01123 has been demonstrated to induce HK2 expression and LDHA expression, thereby enhancing lactate production and promoting the proliferation of tumor cells through the miR-199a-5p/c-Myc axis in NSCLC [31].

#### 2.1.8. HOXA11-AS

Serving as either an oncogene or a tumor-suppressing gene, a newly discovered lncRNA, HOXA11 antisense RNA (HOXA11-AS), plays a vital role in proliferation, invasion, and migration of various cancer cells. Xia et al. demonstrated higher expression levels of HOXA11-AS in NSCLC tissues and cells than in normal ones and showed that HOXA11-AS could promote tumor progression both in vitro and in vivo in NSCLC models [33]. Mechanistically, HOXA11-AS raises the expression of Sal-like protein 4 (SALL4) by functioning as a decoy for miR-3619-5p and subsequently enhancing aerobic glycolysis. However, SALL4, which is a zinc finger transcription factor involved in maintaining the self-renewal of embryonic stem cells and embryonic developmental processes, has been suggested to be highly expressed in LC and was found to participate in NSCLC cell growth and metastasis. Moreover, one study showed that, in cancer cells, SALL4 destabilizes heterochromatin protein-1*α* (HP1*α*), subsequently promoting open chromatin to facilitate the expression of GLUT1 and promote glycolysis [71]. Furthermore, another study showed that SALL4 binds to the promoter region of the HK-2 gene, thus mediating the promotion of glycolysis in gastric cancer cells [72]. Based on these previous studies, SALL4 may have a significant role in glycolysis in LC. However, further investigation is required.

#### 2.1.9. ABHD11-AS1

Rather than functioning as a competing ceRNA or miRNA sponge, the lncRNA ABHD11-AS1 has been reported to regulate gene expression at the transcriptional level. N6-Methyladenosine (m6A) is a methylation modification that can occur on the adenine of RNA, such as mRNA and lncRNA [73]. m6A regulators, such as methyltransferase-like 3 (METTL3), have been reported to execute an m6A-dependent modification of noncoding RNAs (ncRNAs) involved in tumor biological processes [74]. Xue et al. found that m6A methyltransferase METTL3 established the m6A modification and enhanced the stability of the lncRNA ABHD11-AS1 to increase its expression. Furthermore, upregulated ABHD11-AS1 could recruit EZH2 (the pivotal element of the PRC2 complex) to the promoter region of the Krüppel-like factor 4 (KLF4) gene, thereby repressing the transcription of KLF4, ultimately leading to impairment of the Warburg effect [35]. KLF4 is a transcription factor that has an important role in cell differentiation, proliferation, and survival, especially in the context of cancer [75]. A previous study found that enforced KLF4 expression could decrease LDHA expression in cancer cells, thereby forming a KLF4/LDHA signaling pathway to regulate aerobic glycolysis [76].

#### 2.1.10. AL355338

As a highly conserved metalloenzyme that converts 2-phosphoglycerate to phosphoenolpyruvate in glycolysis, alpha-enolase (ENO1) has been universally accepted to play a key role in Warburg effect of various tumor cells [77]. AL355338, functioning as an oncogenic lncRNA, was identified as highly expressed in NSCLC tissues and predicted unfavorable prognosis. Mechanistically, AL355338 was demonstrated to specifically bind to ENO1 and prevent its ubiquitin-mediated degradation to enhance its stability, further facilitating the Warburg effect and other aggressive phenotypes in NSCLC cells [36].

#### 2.1.11. HOTAIRM1

HOTAIRM1 (HOXA transcript antisense RNA myeloid-specific 1), a lncRNA which has been found to be abnormally expressed in various tumors in recent years and dramatically overexpressed in NSCLC tissues, was found to facilitate glycolysis and tumor progression in NSCLC via miR-498 sequestering and ATP binding cassette subfamily E member 1 (ABCE1) enhancement [37, 78].

#### 2.1.12. LINC01537

The expression of LINC01537, a lncRNA remarkably downregulated in LC tissues and cell lines, was observed to positively correlate with survival time and prognosis. Functionally, LINC01537 overexpression enhanced the expression of phosphodiesterase 2A (PDE2A) by stabilizing PDE2A mRNA via RNA-RNA interaction, leading to impaired Warburg effect and energy metabolism [38]. PDE2A, a member of the phosphodiesterase (PDE) family, is an enzyme that degrades cyclic adenosine monophosphate (cAMP) and plays an essential role in mitochondrial respiration [79].

### 2.2. circRNAs

#### 2.2.1. ARHGAP10

Glucose transporter member 1 (GLUT1), an embedded protein on the cell membrane, is responsible for glucose transport across cell membranes. ARHGAP10 expression, which was found to be aberrantly upregulated in NSCLC tissues and cell lines, negatively correlated with the patient's prognosis. Functionally, in NSCLC, ARHGAP10 promotes cell proliferation and metastasis by upregulating GLUT1. Mechanistically, its effect on promoting tumors has been demonstrated to be regulated by the ARHGAP10/miR-150-5p/GLUT1 axis [19].

#### 2.2.2. circMYLK

In addition to GLUT1, another common GLUT type that is aberrantly found in LC is GLUT3, which has been identified as having high affinity for glucose and a high glucose turnover rate [80]. It is strongly expressed in LC; GLUT3 overexpression during stage 1 NSCLC is associated with poor survival [81, 82]. Upregulated GLUT3 in LC promotes glucose import and enhances aerobic glycolysis which induces metabolic reprogramming. Xiong et al. found that a tumor-promoting circRNA, circMYLK, promoted glycolysis and proliferation of NSCLC cells by sponging miR-195-5p and increasing GLUT3 expression [20].

#### 2.2.3. circ_0008928

It has been demonstrated that circ0008928 shows potential to be a decent biomarker for reflecting CDDP sensitivity in NSCLC treatment. Also, circ0008928 could act as an miR-488 sponge to modulate HK2 expression, thus regulating aerobic glycolysis, CDDP resistance, and other malignant biological behaviors, such as cell proliferation, migration, and invasion in NSCLC [21].

#### 2.2.4. circMAGI3

Hepatoma-derived growth factor (HDGF) is a novel growth factor that is involved in numerous cancer processes, including cancer growth, apoptosis, angiogenesis, and metastasis [83]. One study showed that the circRNA, circMAGI3, promoted glycolysis and proliferation of NSCLC by targeting the miR-515-5p/HDGF axis [24]. Furthermore, another study demonstrated that HDGF directly binds to the promoters of GLUT4 and ENO2, thus inducing their expression in cancer and enhancing the Warburg effect [84].

#### 2.2.5. circAGFG1

Another tumor-promoting circRNA in NSCLC is circAGFG1. circAGFG1 is upregulated in NSCLC tissues and cell lines and negatively correlated with the patient's prognosis. Experimentally, circAGFG1 enhanced the malignant behaviors of NSCLC cells by targeting miR-28-5p, with the latter interacting with the 3 untranslated region (3UTR) of HIF-1*α*. Additionally, the enhanced circAGFG1/miR-28-5p/HIF-1*α* axis could subsequently upregulate the expression of key proteins (GLUT1, PGK1, and PKM2) involved in aerobic glycolysis and promote glucose consumption, lactate production, and ATP levels in A549 cells [29].

#### 2.2.6. circSLC25A16

HIF-1*α* is an oxygen-sensitive transcription factor that regulates LDHA transcription in human cancers. Shangguan et al. recently found that the circRNA, circSLC25A16, is significantly upregulated in NSCLC and associated with an unfavorable prognosis. circSLC25A16 increased glucose uptake, lactate production, ATP production, and extracellular acidification rate and promoted proliferation in A549 cells. Mechanistically, circSLC25A16 has been found to function as a ceRNA to enhance the expression of HIF-1*α* via competitively binding with miR-488-3p, thus constructing a circSLC25A16/miR-488-3p/HIF-1*α* signaling pathway. Furthermore, it has been demonstrated that HIF-1*α* could induce LDHA's transcription by directly binding to the LDHA promoter region, which might further account for circSLC25A16's contribution to glycolysis in NSCLC [30].

#### 2.2.7. circ-MEMO1

Ding et al. discovered that high circ-MEMO1 expression is associated with poor prognosis in patients with NSCLC and mainly distributed in the cytoplasmic fraction of NSCLC cells. Experimentally, they observed that circ-MEMO1 knockdown hampered the glycolysis of NSCLC cells, which was evidenced by decreased HK2 and LDHA expression and impaired glucose uptake and lactate production. Furthermore, circ-MEMO1 has been confirmed to sponge miR-101-3p to promote the expression of its target gene, KRAS [32]. Importantly, HK2 is significantly upregulated in oncogenic KRAS-driven NSCLC in mice, which may further explain the positive effect of circ-MEMO1 on glycolysis in NSCLC [59].

#### 2.2.8. circDCUN1D4

Thioredoxin-interacting protein (TXNIP), which plays a significant role in cellular redox balance, has emerged as a vital negative regulator of glucose metabolism in recent years [85]. Located primarily in the nucleus, human antigen R (HuR) is one of the best characterized RNA-binding proteins and plays crucial roles in RNA metabolism including RNA stability [86]. Liang et al. found that circDCUN1D4 is markedly downregulated in lung adenocarcinoma and its expression level is positively associated with better outcomes. Significantly, the binding of HuR and circDCUN1D4 facilitates HuR's transportation to the cytoplasm, thereby promoting the interaction between TXNIP mRNA and the HuR protein and further enhancing TXNIP mRNA's stability. Importantly, circDCUN1D4 was found to suppress glycolysis and metastasis of LC cells by depending on the upregulation of TXNIP expression [39].

#### 2.2.9. circ-ERBB2, circTADA2A, and circ-PGC

Forkhead box M1 (FOXM1) and forkhead box R2 (FOXR2) are both members of the forkhead box transcription factor family. FOXM1 regulates both the G1/S and G2/M phases in the cell cycle and plays a significant role in the pathogenesis of many pulmonary diseases such as LC [87]. FOXR2 was confirmed to directly bind with MYC, one of the well-known oncogenes, and enhance its transcriptional activities to promote tumorigenesis [88]. As tumor-promoting circRNAs, circ-ERBB2 and circTADA2A are both overexpressed in LC tissues and cell lines. Interestingly, they showed similar functions of upregulating FOXM1 by sponging different miRNAs, namely, miR-7-5p and miR-455-3p. Importantly, circ-ERBB2 and circTADA2A have been confirmed to enhance glycolysis and tumorigenesis by targeting the miR-7-5p/FOXM1 and miR-455-3p/FOXM1 axis, respectively [40, 41]. circ-PGC, which was observed to be markedly upregulated in NSCLC tissues and cell lines, has been proven to enhance the Warburg effect and other malignant phenotypes by targeting FOXR2 via miR-532-3p sponging in NSCLC [42].

#### 2.2.10. circPUM1

As a methyltransferase responsible for m6A modification in coding RNAs and ncRNAs, METTL3 can promote the survival and metastasis of human LC cells. Li et al. found that circPUM1, which is aberrantly upregulated in NSCLC tissues and cell lines, facilitates growth and glycolysis in NSCLC cells by sequestering miR-590-5p and enhancing METTL3 expression [43]. METTL3 was demonstrated to induce aerobic glycolysis by regulating the expression of adenomatous polyposis coli (*APC*) or HIF-1*α* in various cancers [89–91].

#### 2.2.11. circ_0000517

Glutamine metabolism has an important role in the citric acid cycle as it replenishes an abundant supply of *α*-ketoglutarate to accelerate glucose metabolism. circ_0000517 has been found to be overexpressed in NSCLC cell lines and NSCLC tissues. Furthermore, circ_0000517 knockdown significantly inhibited the expression of HK2, LDHA, cysteine-preferring transporter 2 (ASCT2), and glutaminase (GLS) in NSCLC cells. The glucose uptake rate, lactate production, and ATP production are markedly impaired in silencing the circ_0000517 group of NSCLC cells. In summary, the study demonstrated that circ_0000517 promoted proliferation and glutamine catabolism of NSCLC cells by interacting with miR-330-5p and subsequently enhancing the expression of Yin yang-1 (YY1) [44]. Additionally, YY1, a GLI-Krüppel class protein with 4 C2H2 zinc finger domains, has been reported to promote aerobic glycolysis in tumor cells and carcinogenesis via GLUT3 transcription activation [92].

#### 2.2.12. circ-PITX1

circ-PITX1 is a recently discovered circRNA that has been demonstrated to contribute to NSCLC progression by functioning as a miRNA sponge [93–95]. Cyclin D2 (CCND2) is an oncogene that encodes proteins in a highly conserved cyclin family. It has been considered a cancer biomarker because of its overexpression in various tumors. Yue et al. confirmed that circ-PITX1 silencing could restrain proliferation, metastasis, cell cycle, glycolysis, and glutamine metabolism in NSCLC. They also established that it is marked by decreased glucose consumption, lactate production, extracellular acidification rate, glutamine consumption, and *α*-KG production. Furthermore, it was observed that circ-PITX1 regulated CCND2 expression by sponging miR-1248 and that this circ-PITX1/miR-1248/CCND2 axis could modulate glycolysis and glutamine metabolism in NSCLC [45].

#### 2.2.13. circ_0006677

Suppressor of cytokine signaling 2 (SOCS2), which is a member of the SOCS protein family, binds with growth factor receptor (GHR) to repress growth factor-induced signal transduction, thus inhibiting cancer progression [96]. circRNA, circ_0006677, which was found to be strikingly downregulated in LC cell lines and tissues, inhibits the malignant biological behavior and glycolysis of NSCLC by increasing SOCS2 expression through ceRNA activity of miR-578 sponging [46].

#### 2.2.14. circNFIX

Metalloprotease domain 10 (ADAM10), a frequently overexpressed protein in human tumors, contributes to malignant progression in many cancers. A study showed that a tumor-promoting circRNA, circNFIX, acts as an miR-212-3p sponge to enhance the malignant progression of NSCLC by upregulating ADAM10. Importantly, glucose uptake and lactate production decreased when circNFIX was knocked down, and this phenomenon could be impaired by miR-212-3p downregulation or ADAM10 overexpression [47].

#### 2.2.15. circ-ENO1

By converting 2-phosphoglycerate to phosphoenolpyruvate, enolase 1 (ENO1) has a crucial role in aerobic glycolysis. An in vivo study has documented that ENO1 promotes tumor invasion and metastasis of LC [97]. Zhou et al. found that circ-ENO1 and its host gene ENO1 were remarkably upregulated in LC cells. Moreover, they confirmed that circ-ENO1 enhances glycolysis to promote proliferation, migration, and epithelial mesenchymal transition and inhibits apoptosis via the circ-ENO1/miR-22-3p/ENO1 axis in LC cells. The tumor-promoting role of circ_ENO1 has been consistently validated in vivo [48].

## 3. Lipid Metabolism

In normal cells, exogenous fatty acids are the major fatty acids involved in lipid metabolism. However, tumor cells rely mostly on the de novo synthesis of fatty acids to support their exaggerated proliferation and other malignant biological behavior despite sufficient exogenous fatty acids in blood circulation [98]. An aberrantly accelerated lipid metabolism was consistently observed in LC [99]. Several key enzymes participating in the de novo synthesis of fatty acids, such as fatty acid synthase (FAS), acetyl-CoA carboxylase (ACC), stearoyl CoA desaturase 1 (SCD1), and ATP citrate lyase (ACLY), were significantly highly expressed in LC [100, 101]. Additionally, researchers found that their overexpression in LC has been markedly correlated with poor prognosis and low survival and is indicative of tumor aggressiveness [102–104].

### 3.1. lncRNA CTD-2245E15.3

ACC1, one of the two subtypes of ACC, is the first key enzyme that catalyzes the transformation of acetyl-CoA to malonyl-CoA during the de novo synthesis of fatty acids. However, the TCA cycle is an important bridge connecting glucose metabolism, lipid metabolism, and amino acid metabolism. As an important intermediate participating in TCA, oxaloacetate needs to be replenished for anabolic uses. This replenishment process, known as anaplerosis, is primarily accomplished through the carboxylation of pyruvate to oxaloacetate via ATP-dependent pyruvate carboxylase (PC) in the context of NSCLC [105]. Wang et al. demonstrated that the novel lncRNA, CTD-2245E15.3, which is mainly located in the cytoplasm and highly expressed in NSCLC and promotes lung carcinogenesis by regulating ACC1 and PC. They observed that CTD-2245E15.3 directly binds to ACC1 and PC, causing reduced phosphorylation of Ser117 in ACC1 and suppressed ubiquitination of PC that lead to enhanced expression and activity of both enzymes. Generally, CTD-2245E15.3 promotes NSCLC cell growth through ACC1-mediated fatty acid synthesis and PC-mediated anaplerosis [49].

### 3.2. lncRNA CCAT1

Intracellular fatty acid-binding proteins (FABPs), which have crucial roles in the uptake and transportation of long-chain fatty acids, are abundantly expressed in almost all tissues in a tissue-specific manner and may be envisioned as prominent regulators of lipid metabolism [106]. As a small member of the FABP family, FABP5 binds natural and synthetic peroxisome proliferator-activated receptor- (PPAR-) *δ* ligands and subsequently mediates the translocation of these ligands to the nucleus to activate PPAR-*δ*. Importantly, this FABP-5/PPAR-*δ* signaling pathway may contribute to tumor progression [107–110]. Chen et al. reported that lncRNA colon cancer-associated transcript 1 (CCAT1), which is remarkably upregulated in lung adenocarcinoma tissues and cell lines, promotes proliferation and angiogenesis in vitro and facilitates carcinogenesis and metastasis in vivo [50]. Functionally, CCAT1 promotes the reprogramming of fatty acid metabolism, as shown by increased lipid accumulation in lung adenocarcinoma cell lines and enhanced expression of several key fatty acid enzymes such as FAS, ACC1, and acyl-CoA oxidase 1. Mechanistically, CCAT1 binds directly with FABP5, facilitating the nuclear translocation of FABP5 to induce the assembly of the PPAR-RXR transcriptional complex on the promoter regions of CD36, VEGFA, and PDK1 and enhance their expression. CCAT1 was confirmed to activate the PI3K/AKT/mTOR signaling pathway in a manner dependent on ubiquitin-specific peptidase 49 and FK506-binding protein 51. In fact, overactivation of the PI3K/AKT signaling pathway has a central role in the dysregulation of lipid metabolism in tumorigenesis by enhancing the expression of enzymes involved in the synthesis of fatty acids and cholesterol [111, 112].

## 4. Glutamine Metabolism

The vigorous life activity of tumor cells is inseparable from accelerated protein synthesis and amino acid metabolism, in which glutamine metabolism plays an important role [113]. As one of the most abundant amino acids in the body, glutamine is the precursor for the synthesis of many amino acids. Glutamine travels across the cell membrane and mitochondrial membrane through amino acid transporter proteins and is then converted to glutamate by glutaminase through deamination. In cancer, because of high levels of transaminase activity, an excess of glutamate is converted to *α*-ketoglutarate to provide sufficient intermediates for the enhanced TCA cycle [114]. Interestingly, in tumor cells (and different from normal cells), mitochondrial respiration is mainly driven by glutamine instead of glucose because glucose is primarily utilized in aerobic glycolysis to produce lactate [115].

### 4.1. circRNA circ-LDLRAD3, lncRNA MINCR, and lncRNA-PVT1-5

Glutamine transporter solute carrier family A1 member 5 (SLC1A5) and glutamine transporter solute carrier family A7 member 5 (SLC7A5) are both amino acid transporter proteins that are aberrantly overexpressed in LC [116]. Researchers have demonstrated that a variant of SLC1A5 is a mitochondrial glutamine transporter that has a critical role in metabolic reprogramming in cancer cells [117]. SLC1A5 and SLC7A5 have been identified as potential therapeutic targets in cancer metabolism because of their significance in tumor metabolism [118, 119].

Xue et al. observed that circ-LDLRAD3 and miR-137 were upregulated and downregulated in NSCLC tissues and cell lines, respectively, and they were both closely associated with clinicopathological features. Essentially, they confirmed that circ-LDLRAD3 regulates SLC1A5 by sponging miR-137 in NSCLC cells, thereby regulating tumor cell proliferation, apoptosis, and mobility [52].

Another study demonstrated that the tumor-promoting lncRNA MINCR promotes proliferation and migration and suppresses apoptosis in NSCLC cells by negatively regulating the miR-126/SLC7A5 axis [53]. Similarly, lncRNA-PVT1-5 also functions as a miRNA sponge and promotes cell proliferation by regulating the miR-126/SLC7A5 signaling pathway in LC [51]. However, glutamine metabolism-related tests were not performed during these studies; therefore, the effects of these ncRNAs on LC glutamine metabolism require further validation.

### 4.2. hsa_circRNA_103809 and circ_0003028

Glutamate oxaloacetate transaminase 1 (GOT1) and glutamate oxaloacetate transaminase 2 (GOT2) are both crucial for promoting tumor progression by regulating glutamate metabolism because transaminases generate *α*-ketoglutarate and indirectly participate in the TCA cycle [120–122]. Zhu et al. found that the novel hsa_circRNA_103809 was overexpressed in cisplatin-resistant NSCLC cells compared to cisplatin-sensitive NSCLC cells. Functionally, overexpression of hsa_circRNA_103809 increased cisplatin resistance in cisplatin-sensitive NSCLC cells by interacting with miR-377-3p and upregulating GOT1 [54].

Furthermore, circ_0003028 was strikingly upregulated in NSCLC tissues and cell lines, and its expression level was positively correlated with malignant biological properties such as colony formation and invasion in NSCLC cells. circ_0003028 directly binds to miR-1298-5p, and GOT2 is a direct target of miR-1298-5p. Taken together, the circ_0003028/miR-1298-5p/GOT2 signaling pathway could be an underlying therapeutic target for NSCLC [55].

## 5. Current Evidence on the Potential Clinical Utility of lncRNAs and circRNAs Involved in Regulating Metabolic Reprogramming of LC as Potential Diagnostic and Prognostic Biomarkers

Till date, there are no lncRNAs or circRNAs that are used clinically as diagnostic or prognostic biomarkers of LC. Nevertheless, the good news is that lncRNAs are being actively explored as potential biomarkers for the diagnosis of LC in a clinical trial, thereby supporting their prevalent link with LC (Clinical Trial Number: NCt03830619). On the other hand, an increasing number of studies have unveiled strong correlation between the expression of lncRNAs or circRNAs and the diagnosis or prognosis of patients with LC.

Using LC tissues, 15 lncRNAs and 17 circRNAs have been shown to be potentially diagnostic biomarkers. The lncRNAs include DUXAP8, IGFBP4-1, BCYRN1, CRYBG3, LINC00243, AC020978, LINC01123, HOXA11-AS, ABHD11-AS1, AL355338, HOTAIRM1, CTD-2245E15.3, CCAT1, MINCR, and PVT1-5 [22, 23, 25, 26, 28, 31, 33–37, 49–51, 53]. The circRNAs comprise circMYLK, circMAGI3, circAGFG1, circSLC25A16, circ-MEMO1, circDCUN1D4, circ-ERBB2, circTADA2A, circ-PGC, circPUM1, circ_0000517, circ-PITX1, circ_0006677, circNFIX, circ-ENO1, circ-LDLRAD3, and circ_0003028 [20, 24, 29, 30, 32, 39–48, 52, 55]. In fact, some of them were also shown to have potential as prognostic indicators in patients with LC; they included DUXAP8, LINC00243, AC020978, LINC01123, HOXA11-AS, ABHD11-AS1, AL355338, circMYLK, circMAGI3, circAGFG1, circSLC25A16, circ-MEMO1, circDCUN1D4, circ-PGC, circPUM1, circ_0000517, circ_0006677, circNFIX, CCAT1, and MINCR [20, 22, 24, 26, 29–36, 39, 42–44, 46, 47, 50, 53]. Besides, two other lncRNAs, LINC01537 and ARHGAP10, were only found to have potential as prognostic indicators [19, 38].

Clinically, the expression of IGFBP4-1, ABHD11-AS1, circMAGI3, circSLC25A16, circDCUN1D4, and circPUM1 in LC tissues was associated with TNM stage; the expression of DUXAP8, circ_0000517, and circ-LDLRAD3 correlated with TNM stage and LNM; while the expression of AC020978, HOXA11-AS, circ-PGC, and circ_0006677 was associated with TNM stage, LNM, and tumor size [22–24, 26, 30, 33, 35, 39, 42–44, 46, 52]. In addition, CRYBG3 expression correlated with distant metastasis; CCAT1 expression correlated with clinical stage; circNFIX expression was associated with TNM stage and distant metastasis; circ-MEMO1 expression correlated with clinical stage and LNM; circMYLK was associated with TNM stage and tumor size; LINC01123 expression was associated with TNM stage, T stage, and LNM, while AL355338 expression was associated with TNM stage, T stage, LNM, and distant metastasis [20, 28, 31, 32, 36, 47, 50].

Most of these ncRNAs in LC diagnostic or prognostic prediction were found to be aberrantly upregulated and functioning as cancer-causing molecules; from Kaplan-Meier analysis, their expression correlates with the poor survival outcomes of patients. However, there are three remarkably downregulated ones in LC: LINC01537, circDCUN1D4, and circ_0006677. It is worth noting that in contrast to other “bad” ncRNAs discussed in this review, the expression of the three in LC was all observed to positively correlate with better survival outcomes of the patients [38, 39, 46]. Although LC tissue was the sample type detected in majority of the researches included in the review, two circRNAs, circ_0008928 and circAGFG1, were reported to be deregulated in the serum of LC patients, which make them potential noninvasive circulating biomarkers for LC diagnosis or prognosis [21, 29]. circ_0008928 was found to be significantly overexpressed in serum exosomes of patients with cisplatin-resistant NSCLC compared to those with cisplatin-sensitive NSCLC, making it a potential diagnostic biomarker in cisplatin-resistant NSCLC [21]. Besides, circAGFG1 expression was found to be greatly upregulated in both serum samples and LC tissues of patients with NSCLC; also, it was confirmed to be a potential diagnostic and prognostic biomarker for poor survival outcomes [29].

Examples of lncRNAs and circRNAs regulating metabolic reprogramming of LC and their potential in LC diagnostic and prognostic prediction are listed in Table 2.

↑: upregulated; ↓: downregulated; RT-qPCR: real-time quantitative polymerase chain reaction; ISH: in situ hybridization; FISH: fluorescence in situ hybridization; TNM stage: tumor node metastasis stage; LNM: lymph node metastasis; OS: overall survival; DFS: disease-free survival.

## 6. Future Perspectives regarding lncRNAs and circRNAs as Novel Therapeutic Targets for LC

Apart from diagnostic and prognostic biomarkers, the clinical potential of lncRNAs and circRNAs in LC is also dependent on their therapeutic value. Some preclinical studies have reported their potential metabolism-associated roles in mechanisms of drug action and drug resistance. Hua et al. found that the lncRNA X-inactive-specific transcript could contribute to the cisplatin resistance of LC cells by sponging miR-101-3p in a glycolysis-dependent manner [123]. Propofol, an anesthetic extensively used intravenously during surgery, was recently found to suppress LC tumorigenesis and aerobic glycolysis through both the circ-ERBB2/miR-7-5p/FOXM1 and circTADA2A/miR-455-3p/FOXM1 axes [40, 41]. Ma et al. demonstrated that hsa_circ_0002130 facilitates osimertinib resistance and the Warburg effect in NSCLC via the downregulation of miR-498.

However, the practical applications of lncRNAs and circRNAs in LC therapy are still in their infancy. Although there have been some in vivo studies on the exploration of therapeutic delivery strategies for miRNAs in LC, no in vivo studies on lncRNA-based or circRNA-based therapeutic strategies for LC have emerged, and the clinical development of their therapeutics has not been reported [124–128]. In fact, the development of ncRNA-based therapy involves three major hurdles: specificity, delivery, and tolerability [127, 129]. Therefore, minimized off-target effects, stable and efficient delivery vehicles, and attenuated or even nonexistent immune rejection are the goals that researchers should pursue in the field of lncRNA-based and circRNA-based therapy for LC.

As the key to ncRNA therapeutics, the delivery system has undergone significant development in recent years. To date, approximately four major categories have been reported as classical carriers transporting lncRNAs or circRNAs during either in vitro or in vivo studies; they include viral vectors, liposomes, nanoparticles, and exosomes [129, 130]. Viral vectors, which have high transfection efficiency and an enduring expression time, are predominantly utilized in basic research rather than clinical practice because of controversy regarding their safety. Liposomes, especially nanoliposomes, are gaining increasing attention during preclinical studies because of their reduced toxicity, immunogenicity, and other desirable advantages [131]. Liposomes have been frequently used to deliver lncRNAs during preclinical trials targeting LC and for other cancer therapies [132–134]. Additionally, nanoparticle delivery can overcome many limitations of small interfering RNAs targeting ncRNAs by protecting them from degradation, facilitating cellular uptake, and preventing immune activation [127]. Unlike these previously described vectors, which are produced in vitro through chemical or biological synthesis, exosomes are naturally secreted by living cells. Therefore, patient-derived tissues may become individualized and biocompatible sources of ncRNA-packaged exosomes. It is pertinent to note that surface modification of exosomes, especially specific peptides that bind their receptors within the target cell, effectively minimizes the off-target effect [135, 136]. Moreover, exosome-transmitted lncRNAs and circRNAs have had remarkable effects on tumor progression in LC during preclinical experiments [137–139]. Although there are some potentially effective lncRNA-based and circRNA-based delivery strategies, their applications have only been explored in a few animal experiments. Therefore, the efficacy and side effects of each delivery strategy need to be comprehensively studied and evaluated in more extensive fundamental experiments and clinical trials.

## 7. Conclusions

Although an increasing number of studies have recently elucidated ncRNAs in LC and made some important progress, studies on the specific roles of lncRNAs and circRNAs in LC metabolism are limited. Therefore, further investigation is necessary. However, metabolism-oriented drug therapy for LC based on lncRNAs or circRNAs, such as propofol, should be further studied because of the lack of clinical data indicating their efficacy and safety [40, 41]. In fact, miRNA-based therapies such as miRNA mimics and anti-miRs are in phase II or phase III of clinical development, but no lncRNA-based or circRNA-based therapeutics are being used in the clinic at this time. We hope that more lncRNA-based and circRNA-based studies targeting tumor metabolism will emerge that provide us with a better understanding of the mechanisms of LC carcinogenesis and development and help with the development of strategies for personalized treatment.

## Figures and Tables

**Table 1:** List of lncRNAs and circRNAs that participate in lung cancer metabolic reprogramming.

Metabolism	lncRNA/circRNA	Deregulation	Target miRNA	Downstream component	Function	Reference
Glucose metabolism	ARHGAP10	↑	miR-150-5p	GLUT1	Enhances Warburg effect	[19]
	circMYLK	↑	miR-195-5p	GLUT3	Enhances Warburg effect	[20]
	circ_0008928	↑	miR-488	HK2	Enhances Warburg effect	[21]
	DUXAP8	↑	miR-409-3p	HK2, LDHA	Enhances Warburg effect	[22]
	IGFBP4-1	↑	—	HK2, LDHA, PDK1	Enhances Warburg effect	[23]
	circMAGI3	↑	miR-515-5p	HDGF	Enhances Warburg effect	[24]
	BCYRN1	↑	miR-149	PKM2	Enhances Warburg effect	[25]
	AC020978	↑	—	PKM2, HIF-1*α*	Enhances Warburg effect	[26]
	KIMAT1	↑	—	LDHB	Enhances Warburg effect	[27]
	CRYBG3	↑	—	LDHA	Enhances Warburg effect	[28]
	circAGFG1	↑	miR-28-5p	HIF-1*α*	Enhances Warburg effect	[29]
	circSLC25A16	↑	miR-488-3p	HIF-1*α*	Enhances Warburg effect	[30]
	LINC01123	↑	miR-199a-5p	c-Myc	Enhances Warburg effect	[31]
	circ-MEMO1	↑	miR-101-3p	KRAS	Enhances Warburg effect	[32]
	HOXA11-AS	↑	miR-3619-5p	SALL4	Enhances Warburg effect	[33]
	LINC00243	↑	miR-507	PDK4	Enhances Warburg effect	[34]
	ABHD11-AS1	↑	—	KLF4	Enhances Warburg effect	[35]
	AL355338	↑	—	ENO1	Enhances Warburg effect	[36]
	HOTAIRM1	↑	miR-498	ABCE1	Enhances Warburg effect	[37]
	LINC01537	↓	—	PDE2A	Inhibits aerobic glycolysis	[38]
	circDCUN1D4	↓	—	TXNIP	Inhibits aerobic glycolysis	[39]
	circ-ERBB2	↑	miR-7-5p	FOXM1	Enhances Warburg effect	[40]
	circTADA2A	↑	miR-455-3p	FOXM1	Enhances Warburg effect	[41]
	circ-PGC	↑	miR-532-3p	FOXR2	Enhances Warburg effect	[42]
	circPUM1	↑	miR-590-5p	METTL3	Enhances Warburg effect	[43]
	circ_0000517	↑	miR-330-5p	YY1	Enhances Warburg effect	[44]
	circ-PITX1	↑	miR-1248	CCND2	Enhances Warburg effect	[45]
	circ_0006677	↓	miR-578	SOCS2	Inhibits aerobic glycolysis	[46]
	circNFIX	↑	miR-212-3p	ADAM10	Enhances Warburg effect	[47]
	circ-ENO1	↑	miR-22-3p	ENO1	Enhances Warburg effect	[48]

Lipid metabolism	CTD-2245E15.3	↑	—	ACC1, PC	Enhances fatty acid synthesis and anaplerosis	[49]
	CCAT1	↑	—	FAS, ACC1, ACOX1, PDK1, CD36, VEGFA, and PI3K/AKT/mTOR signaling pathway	Enhances fatty acid synthesis	[50]

Glutamine metabolism	PVT1-5	↑	miR-126	SLC7A5	Promotes cell proliferation	[51]
	circ-LDLRAD3	↑	miR-137	SLC1A5	Promotes cell proliferation and mobility and supresses apoptosis	[52]
	MINCR	↑	miR-126	SLC7A5	Promotes cell proliferation and migration and suppresses apoptosis	[53]
	hsa_circRNA_103809	↑	miR-377-3p	GOT1	Increases cisplatin resistance	[54]
	circ_0003028	↑	miR-1298-5p	GOT2	Promotes cell proliferation, colony formation, cell cycle progression, migration and invasion and suppresses apoptosis and autophagy	[55]

↑: upregulated; ↓: downregulated; ACC1: acetyl-CoA carboxylase 1; ACOX1: acyl-coenzyme A oxidase 1; ADAM10: metalloprotease domain 10; CCND2: cyclin D2; circRNAs: circular RNAs; ENO1: *α*-enolase; FAS: fatty acid synthase; FOXM1: forkhead box M1; GOT1: glutamate oxaloacetate transaminase 1; GOT2: glutamate oxaloacetate transaminase 2; GLUT 1: glucose transporter member 1; GLUT 3: glucose transporter member 3; HDGF: hepatoma-derived growth factor; HIF-1*α*: hypoxia-inducible factor 1-alpha; HK2: hexokinase 2; KLF4: Krüppel-like factor 4; LDHA: lactate dehydrogenase A; LDHB: lactate dehydrogenase B; lncRNAs: long noncoding RNAs; METTL3: methyltransferase like 3; PC: pyruvate carboxylase; PDK1: pyruvate dehydrogenase kinase 1; PKM2: pyruvate kinase 2; PDK4: pyruvate dehydrogenase kinase 4; SALL4: Sal-like protein 4; SLC1A5: glutamine transporter solute carrier family A1 member 5; SLC7A5: glutamine transporter solute carrier family A7 member 5; TXNIP: thioredoxin-interacting protein; VEGFA: vascular endothelial growth factor A; YY1: Yin yang-1; ABCE1: ATP binding cassette subfamily E member 1; FOXR2: forkhead box R2; PDE2A: phosphodiesterase 2A.

**Table 2:** Summary of lncRNAs and circRNAs involved in the metabolic reprogramming of LC as potential diagnostic or/and prognostic biomarkers.

lncRNA/circRNA	Deregulation	Detection method	Sample type and the number of samples	Survival analysis and the number of patients enrolled	Survival outcomes correlated with the expression	Clinical characteristics associated with the expression	Biomarker category	Reference
DUXAP8	↑	RT-qPCR	LC tissues; 66	OS; 66	Poor survival outcome	TNM stage, LNM	Diagnosis/prognosis	[22]
IGFBP4-1	↑	RT-qPCR	LC tissues; 159	—	—	TNM stage	Diagnosis	[23]
BCYRN1	↑	RT-qPCR	LC tissues; 20	—	—	—	Diagnosis	[25]
CRYBG3	↑	RT-qPCR	LC tissues; 23	—	—	Distant metastasis	Diagnosis	[28]
LINC00243	↑	RT-qPCR	LC tissues; 59 from TCGA database and 70 from the patients enrolled	OS; 70	Poor survival outcome	—	Diagnosis/prognosis	[34]
AC020978	↑	FISH, RT-qPCR	LC tissues; 92(FISH), 16(RT-qPCR)	OS; 92	Poor survival outcome	TNM stage, LNM, tumor size	Diagnosis/prognosis	[26]
LINC01123	↑	RNA-seq, FISH, RT-qPCR	NSCLC tissues; 3 (RNA-seq), 92 (FISH), and 16 (RT-qPCR)	OS; 92	Poor survival outcome	TNM stage, T stage, LNM	Diagnosis/prognosis	[31]
HOXA11-AS	↑	RT-qPCR	LC tissues; 40	OS; 40	Poor survival outcome	TNM stage, tumor size, LNM	Diagnosis/prognosis	[33]
ABHD11-AS1	↑	RT-qPCR	LC tissues; 40	OS; exact number not mentioned	Poor survival outcome	TNM stage	Diagnosis/prognosis	[35]
AL355338	↑	FISH, RT-qPCR	LC tissues; 80 (RT-qPCR)	OS; 80	Poor survival outcome	TNM stage, T stage, LNM, distant metastasis	Diagnosis/prognosis	[36]
HOTAIRM1	↑	RT-qPCR	NSCLC tissues (exact number not mentioned)	—	—	—	Diagnosis	[37]
LINC01537	↓	RNA-seq	LC tissues; 8	Prognostic score in the TCGA LC cohort	Better survival outcome	—	Prognosis	[38]
ARHGAP10	↑	FISH	LC tissues; 92	OS; exact number not mentioned	Poor survival outcome	—	Prognosis	[19]
circMYLK	↑	RT-qPCR	LC tissues; 103	OS; 103	Poor survival outcome	TNM stage, tumor size	Diagnosis/prognosis	[20]
circ_0008928	↑	RT-qPCR	Serum exosomes; 28 from cisplatin-sensitive NSCLC patients and 19 from cisplatin-resistant NSCLC patients	—	—	—	Diagnosis	[21]
circMAGI3	↑	RT-qPCR	LC tissues; 30	OS; exact number not mentioned	Poor survival outcome	TNM stage	Diagnosis/prognosis	[24]
circAGFG1	↑	RT-qPCR	NSCLC tissues and corresponding serum samples; 45	OS; 45	Poor survival outcome	—	Diagnosis/prognosis	[29]
circSLC25A16	↑	RT-qPCR	NSCLC tissues; 40	OS; exact number not mentioned	Poor survival outcome	TNM stage	Diagnosis/prognosis	[30]
circ-MEMO1	↑	RT-qPCR	NSCLC tissues; 52	OS; 52	Poor survival outcome	Clinical stage, LNM	Diagnosis/prognosis	[32]
circDCUN1D4	↓	ISH	NSCLC tissues; 92	OS; exact number not mentioned	Better survival outcome	TNM stage	Diagnosis/prognosis	[39]
circ-ERBB2	↑	RT-qPCR	LC tissues; 31	—	—	—	Diagnosis	[40]
circTADA2A	↑	RT-qPCR	LC tissues; 30	—	—	—	Diagnosis	[41]
circ-PGC	↑	RT-qPCR	NSCLC tissues; 33	OS; exact number not mentioned	Poor survival outcome	TNM stage, tumor size, LNM	Diagnosis/prognosis	[42]
circPUM1	↑	RT-qPCR	NSCLC tissues; 62	OS; 62	Poor survival outcome	TNM stage	Diagnosis/prognosis	[43]
circ_0000517	↑	RT-qPCR	NSCLC tissues; 60	OS; 60	Poor survival outcome	TNM stage, LNM	Diagnosis/prognosis	[44]
circ-PITX1	↑	RT-qPCR	NSCLC tissues; 41	—	—	—	Diagnosis	[45]
circ_0006677	↓	RT-qPCR	NSCLC tissues; 88	OS; 88	Better survival outcome	TNM stage, tumor size, LNM	Diagnosis/prognosis	[46]
circNFIX	↑	RT-qPCR	NSCLC tissues; 55	OS; 55	Poor survival outcome	TNM stage, distant metastasis	Diagnosis/prognosis	[47]
circ-ENO1	↑	RT-qPCR	LC tissues; 64	—	—	—	Diagnosis	[48]
CTD-2245E15.3	↑	RT-qPCR	NSCLC tissues from TCGA database; 535	—	—	—	Diagnosis	[49]
CCAT1	↑	ISH, RT-qPCR	LC tissues; 10	OS/DFS, 504	Poor survival outcome	Clinical stage	Diagnosis/prognosis	[50]
circ-LDLRAD3	↑	RT-qPCR	NSCLC tissues; 60	—	—	TNM stage; LNM	Diagnosis	[52]
MINCR	↑	RT-qPCR	NSCLC tissues; 35	OS; exact number not mentioned	Poor survival outcome	—	Diagnosis/prognosis	[53]
lncRNA-PVT1-5	↑	RT-qPCR	LC tissues; 80	—	—	—	Diagnosis	[51]
circ_0003028	↑	RT-qPCR	NSCLC tissues; 53	—	—	—	Diagnosis	[55]
